# The study of vancomycin use and its adverse reactions associated to patients of a brazilian university hospital

**DOI:** 10.1186/1756-0500-4-236

**Published:** 2011-07-15

**Authors:** Daniel Savignon Marinho, Gisele Huf, Bruno LA Ferreira, Helena Castro, Carlos R Rodrigues, Valeria Pereira de Sousa, Lúcio M Cabral

**Affiliations:** 1 Departamento de Medicamentos, Faculdade de Farmácia, UFRJ, Rio de Janeiro, RJ, Brazil; 2 Instituto Nacional de Controle de Qualidade em Saúde, Fundação Oswaldo Cruz, RJ, Brazil; 3 LABioMol, Instituto de Biologia, UFF, Niterói, RJ, Brazil

## Abstract

**Background:**

Vancomycin is an antibiotic of growing importance in the treatment of hospital infections, with particular emphasis on its value in the fight against methicillin-resistant *Staphylococcus aureus*. However its usage profile must be evaluated to assure maximum benefit and minimum risk.

**Findings:**

A cross-sectional retrospective study was carried out among inpatients that received vancomycin in a Brazilian quaternary hospital. The occurrence of adverse reactions reported was evaluated in medical records relating to patients taking vancomycin during a one year period. Males comprised 52% (95% CI: 41.7-60.2%) of the sample population, with a mean age of 50.6 (95% CI: 47.2-54.0) years and mean treatment period of 9.7 (95% CI: 8.0-11.5) Days. It was verified that nephrotoxicity occurred in 18.4% (95% CI: 11.3-27.5) of patients, Red man syndrome occurred in 2% (95% CI 0.2-7.2), while the occurrence of thrombocytopenia was 7.1% (95% CI: 2.9-14.2).

**Conclusions:**

It may be noted that even after 50 years of use, adverse reactions associated with vancomycin continue with high frequency, presenting a public health problem, especially considering its current use in cases of multidrug resistant infections. In this context, we emphasize the importance of intensive pharmacovigilance in hospital as a surveillance tool after drug approval by the sanitary authority.

## Findings

### Introduction

Vancomycin is a complex glycopeptide antibiotic with an unusual structure and molecular mass of approximately 1500 Da. Vancomycin acts primarily inhibiting the cell-wall biosynthesis. Its action against gram-positive cell walls is related to the prevention of the incorporation of N-acetylmuramic acid and N-acetylglucosamine into the peptideoglycan matrix. Its therapeutic use against infections caused by gram-positive bacteria started in the 1950's and it is still used currently. During the 1960's and 70's its use suffered a large decline, due to the high incidence of adverse reactions and the introduction of semisynthetic penicillins with broadest spectrum of action in the therapeutic arsenal [1]. However, the rapid development of bacterial resistance to semisynthetic penicillin, especially methicillin-resistant *Staphylococcus aureus *(MRSA), led to the return of the use of vancomycin on a large scale from the 1980's.

During the following two decades, some studies showed a possible link  between the widespread use of vancomycin and a rise in the incidence of  adverse reactions, especially nephrotoxicity [1], ototoxicity [2], thrombocytopenia [3,4], epidermal necrosis [5], neutropenia [6], phlebitis [7], and a pathology related to histamine release known as the red man syndrome [8-10], which highlights the need for more effective monitoring of the use of this antibiotic. The reports of *Enterococcus faecalis *resistant to vancomycin published since the 1990's, and recently in Brazilian hospitals, further emphasizes the need to rationalize the use of this drug [11]. Others studies have been carried out in order to confirm the safe use of vancomycin, as the recent systematic review that compared vancomycin safety and efficacy with the other available glycopeptide [12]. This study showed the importance of producing more scientific data on vancomycin ADR. Nevertheless, the severity of these effects is reflected in the constant concern with the monitoring techniques employed and vancomycin's effectiveness when it is used [13]. Therefore, this study evaluates the use of vancomycin and the prevalence of the main adverse reactions in a large university hospital in quaternary care.

### Methods

#### Population studied

The retrospective cross-sectional study population consisted of individuals who had used vancomycin during the period of July 2003 to June 2004, as this data were the most reliable in relation to the storage process and accessible at the time of the study, while attending a university hospital in Brazil. From the total of 350 patients that utilized vancomycin during the study period, a random sample was selected based on the sample size estimation calculated by statcalc tool from epiinfo^® ^(version 3.3.2), utilizing 95% of precision. The estimated sample size was 122 patients. Based on that estimate, a random sample of 131 patients was selected as the population for this study. After applying the exclusion criteria, 98 medical records were included for analysis. The study included all individuals older than 18 years, with use of vancomycin confirmed by documentation in their medical records. It excluded mainly those individuals whose records contained insufficient laboratory data to allow assessment of presence or absence of adverse reactions.

#### Definitions

For measuring and comparing the data regarding the exposure of vancomycin, we utilized as reference the mean daily dose of the drug when used for its main indication, being equal to 2 g of vancomycin [6]. Thus, all the vancomycin exposure data were expressed as grams of vancomycin. Adverse reactions were categorized as: probable (event or laboratory test abnormality, with reasonable time relationship to drug intake, unlikely to be attributed to disease or other drugs, response to withdrawal clinically reasonable, rechallenge not required); possible (event or laboratory test abnormality, with reasonable time relationship to drug intake, could also be explained by disease or other drugs, information on drug withdrawal may be lacking or unclear); conditional (event or laboratory test abnormality, more data for proper assessment needed, or additional data under examination); and uncertain (event or laboratory test abnormality, with a time to drug that makes a relationship improbable, but not impossible, disease or other drugs provide plausible explanations) [14-16].

#### Ethics

In order to comply with regulations relating to Human Research, requirements regarding the confidentiality and secrecy of the information collected were adhered to. The study was approved by the ethics committee of the Faculty of Medicine of Federal University of Rio de Janeiro, being filed under the number 115/04. The project was approved on 9 September 2004.

#### Data Collection

A survey was structured through the analysis of the data from the hospital IT system and the reports of the use of antimicrobials archived by the committee of hospital infection control, only standardized forms were used for data collection, and the following variables referenced in the literature were selected as relevant to the occurrence of adverse drug reaction: age [17], sex, serum creatinine [17], use of nephrotoxic drugs [18,19], hypoalbuminemia [19], dose and reason for use. Signs and symptoms of adverse reactions were evaluated using the criteria in Table 1.

**Table 1:** Criteria followed for evaluate signs and symptoms of adverse reactions

Adverse symptoms/reactions	Source	Criteria
Phlebitis	Medical records	Indicated by medical personnel

Nephrotoxicity	Medical records/Laboratorial analysis	Serum creatinine concentrations of 0.5 mg/dl or 50% increase over the baseline, whichever is the higher value^14^

Neutropenia	Medical records/Laboratorial analysis	Neutrophil count below 1,000 cells per cm^3 ^of peripheral blood^1^

Ototoxicity	Medical records	Indicated by medical personnel after audiometric evaluations and reports of hearing loss after treatment, obtained by attending physicians in the process of anamnesis;

Thrombocytopenia	Medical records/Laboratorial analysis	Platelet count less than 100,000 per microliter of peripheral blood

Red man syndrome	Medical records	Indicated by attending physicians and reviewers, faced with the following standard of diagnosis: flushing, itching, chest pain, muscle spasm or hypotension during infusion of vancomycin

The standardized forms contained the following information: Patient ID; date of birth; body weight; height; sex; reason to utilize vancomycin; date of use; daily dose; length of infusion; previous use of vancomycin; laboratory data (total serum proteins; serum albumin; hematocrit; total white blood cells count; neutrophils count; serum creatinine; platelets); other drugs; ADR description; concomitant diseases. The data were collected by one researcher and checked by another to maintain the quality of form filling.

As the cross-sectional design of study has not the ability to establish causality relationship, we screened every detected ADR with two distinct algorithms Karch and Lasagna algorithm and Naranjo & col algorithm, both previously published and already established to classify the probability of occurrence of an ADR, minimizing the risk of bias during the ADR detection process [14,15].

#### Data analysis

The analysis was performed by comparing the prevalence of adverse reactions between the various strata of this subpopulation [20,21]. The database created to manage the information collected during the hospital records search was set using the epiinfo software version 3.3.2 (Centers for Disease Control and Prevention - CDC, USA). The prevalence ratios were calculated by comparing the prevalence of ADR in groups of patients that were/were not exposed to specific risk factors (other drugs, previous vancomycin use, and others). These prevalence ratios were calculated using epiinfo^® ^and compared utilizing chi square or the fisher exact test.

## Results and Discussion

### Prevalence of Adverse Reactions for Vancomycin

The prevalence of adverse reactions to vancomycin observed in medical records is presented in Table 2. From the data collected it was found that 27.6% (95% CI: 19.0 - 37.5%) of the patients treated with vancomycin had some of the adverse reactions classically triggered by the use of this drug [2]. The prevalence of ADR to vancomycin is alarming as these reactions may worsen the clinical condition of patients which can often already be grave and, in some cases, enhance the adverse effects of other drugs. A direct comparison is difficult to be made between the total prevalence of adverse reactions in the present study and those in other previous reports, since most previous reports set their focus on a specific adverse reaction and not on a range of possible adverse reactions associated with this drug.

**Table 2:** Prevalence of adverse reactions to vancomycin during the study period

Type of adverse reaction	Number of patients	Percentage	95% CI (%)
Nephrotoxicity	18	18.4	11.3 - 27.5
Neutropenia	2	2	0.2 - 7.2
Ototoxicity	1	1	0.0 - 5.6
Red Man Syndrome	2	2	0.2 - 7.2
Thrombocytopenia	7	7.1	2.9 - 14.2

The frequency of renal ADR of 18.4% (95% CI: 11.3 - 27.5%) was similar to the study of Farber & col, that observed approximately 15% of patients suffering renal adverse reactions [2]. The prevalence of renal ADR observed is closer to the data for elderly patients, over 60 years, presented by Vance-Bryan [17]. The study published by Rybak & col [19], establishes a prevalence of approximately 10% for this type of ADR. However, it should be noted that the average age of the patients in our study was around 50 years. The prevalence of this ADR is even more important because it may impact on other drugs with primarily renal elimination and also the loss in renal clearance capacity may increase vancomycin drug plasma levels [22,23], as it is mainly eliminated by renal filtration, worsening the ADR. This fact reinforces the importance of laboratory monitoring vancomycin use [13].

A significant finding of this study was that the prevalence of thrombocytopenia was 7.1% (95% CI: 2.9 - 14.2%). This value was discrepant with the fact that thrombocytopenia has historically been regarded as a rare adverse reaction to vancomycin [3,24]. Farber & col. study do not even cite this ADR among those observed [2]. The prevalence of neutropenia was 2% (0.2 - 7.2%) being consistent with studies carried out previously [2] including one cited by Rocha & col [7], where both report an incidence close to 2%. It should be noted that many of the neutropenia-type reactions in patients studied in this research were excluded as ADRs because patients were also using antineoplastic drugs that may more frequently lead to the development of this ADR [8], however case reports have increased [3,7] and greater attention has been given to this ADR. In a retrospective study with pediatric patients [25], thrombocytopenia corresponded to 26.3% of ADRs, and was the second most prevalent ADR to vancomycin in that study. More recently, the incidence of this ADR was studied by monitoring patients treated with vancomycin over a five year period and this corroborated the results reported in the present study [26].

As the ADR most characteristic of vancomycin use, the RMS, has received most attention in studies that report the use of this drug. In the present study, the prevalence of this ADR was 2% (95% CI: 0.2 - 7.2%), a value quite close to that found by Farber & col [2], which was 3%. The retrospective study of children by Hing & col [23] had a prevalence of about 2% for this ADR, in addition to the study of Wallace & col [9], which established the prevalence as 0-35% in patients and 70-90% in healthy volunteers. Considering that the occurrence of RMS is directly related to the rate of infusion of vancomycin [20,22,23,27,28], the fact that 60% of patients who used vancomycin had no information in their records regarding the duration of infusion of vancomycin solution shows that there is a dangerous lack of concern or ignorance of this type of ADR and the recommended method of drug administration.

Ototoxicity is an ADR that had a prevalence of 1% (95% CI: 0 - 5.6%) with similar data presented by Farber & col. This study has no recorded occurrence of phlebitis. However the study of Farber & col [2] showed a prevalence of 13% of this ADR, being the second most prevalent. In the study by Hing & col [23] it is presented, in conjunction with pain, as the most prevalent ADR affecting 11% of patients treated with vancomycin. A plausible reason for the low prevalence of ototoxicity is the non-completion of routine audiometric tests in hospitalized patients, which was the real measure of prevalence of this ADR [2].

### Sample Characteristics and Vancomycin Exposure

Of the 131 selected patients, 98 were effectively analyzed, as the medical charts of 31 patients had inadequate records, especially regarding to the clinical laboratory data, becoming impossible the verification of signs and symptoms of ADR, and 2 patients were aged less than 18 years, being therefore excluded from the study. Table 3 shows the main characteristics of the population studied and no evidence of clustering in the extremes of age or by sex, which could suggest confounding factors for the prevalence estimate of ADR [16,17].

**Table 3:** Characteristics of patients evaluated

Characteristics	Median	Mean	Mean 95% CI (%)
Age (years)	51.5	50.6	47.2 - 54.0
Treatment time (days)	7.0	9.7	8.0 - 11.5
Total Vancomycin dose (g)	24	18.3	12.4 - 23.8
Vancomycin Daily dose (g/day)	1.9	1.9	1.6 - 2.4
Initial creatinine (mg/dl)	1.0	2.0	1.5 - 2.5
Albumin (g/dl)	1.8	1.9	1.6 - 2.1

Statistical analysis was performed using Student's t test.

The data analysis (Table 4) of initial creatinine and serum albumin showed that patients that presented with ADRs did not show any laboratory data at the beginning of treatment that would have identified them as a group at risk of suffering adverse reactions [29].

**Table 4:** Characteristics of patients that did and did not presented adverse drug reactions (ADRs)

Characteristics		Patients ADR +			Patients ADR -	
	Median	Mean	95%CI(%)	Median	Mean	95% CI(%)
**Age (years)**	47	49.1	42.0 - 56.3	52	51.1	47.3 - 55.0
**Treatment time (days)**	7.0	11.6	7.7 - 15.5	7.0	9.0	7.1 - 10.9
**Total Vancomycin dose (g)**	8.0	23.3	6.5 - 40.1	8.0	16.2	11.2 - 21.2
**Vancomycin Daily dose (g/day)**	1.4	2.0	1.1-2.8	1.0	1.9	1.5-2.4
**Initial creatinine (mg/dl)**	1.1	1.7	1.1 - 2.3	1.0	2.1	1.5 - 2.8
**Albumin (g/dl)**	1.4	1.9	1.6 - 2.1	1.75	1.9	1.6 - 2.1

Statistical analysis was performed using z-test. P > 0.05 for all the parameters evaluated comparing patients ADR + and -.

The main reasons for the use of vancomycin among the studied patients were sepsis 12.2% (95% CI: 6.5 - 20.4%), nosocomial pneumonia 12.2% (95% CI: 6.5 - 20.4%), catheter-associated infection 11.2% (95% CI: 5.7 - 19.2%) and blood culture with Gram positive cocci 11.2% (95% CI: 5.7 - 19.2%). Among patients who had some type of adverse reaction associated with vancomycin, the main reasons for using the drug were septic shock 11.1% (95% CI: 2.4 - 29.2%), nosocomial pneumonia 11.1% (95% CI: 2.4 - 29.2%), endocarditis 7.4% (95% CI: 0.9 - 24.3%) and abdominal sepsis 7.4% (95% CI: 0.9 - 24.3%).

The analysis of the total doses of vancomycin presented in Table 4 shows that there is no significant difference in total dose of vancomycin administered to the group of patients that did present with an ADR compared to those that did not (p >0.05). The analysis of average daily dose of vancomycin also showed no significant difference between the average daily dose given to patients that did and those that did not present with an ADR (p >0.05). Statistical analysis comparing the subgroups of ADR+ and ADR- patients as presented at Table 4 did not present any statistically significant difference (P > 0.05).

Concurrent use of other antimicrobial drugs was also evaluated, since some studies suggest synergism in their ability to cause adverse reactions with vancomycin [18,19,26,29,30]. It was found that cefepime, imipenem/cilastatin and ciprofloxacin were the most commonly used antibiotics in both groups of patients, those that did and did not present with an ADR. Broad-spectrum beta-lactam drugs were the most frequent antimicrobial drugs used in both groups. The prevalence ratio was calculated as an indicator of the synergistic effect of these antibiotics, but none had a statistically significant relation.

The rate of infusion of vancomycin is one of the factors classically associated with the occurrence of adverse reactions [20]. It is recommended that the duration of the solution infusion is not less than 60 min. Thus, the duration of vancomycin solution infusion was included in the analysis of the factors possibly associated with ADRs, and this was categorized as greater than 60 min, less than 60 min or without any record. However it was not possible to ascertain any statistically significant relationship between duration of infusion and the occurrence of ADR. Another worrying fact was the absence of records of the infusion rate of vancomycin solution in 62.24% of the prescriptions of the total population, a fact that suggests that little importance is assigned to the scientific evidence of a higher incidence of cutaneous and systemic adverse reactions in patients with infusion of vancomycin over periods of less than 60 min [8,20,22].

This study is retrospective and only the adverse effects reported in medical records could be evaluated. The absence of data in the medical records could reflect a practice of not reporting some types of ADR, considered as a routine part of the patient condition, so that there is a low prevalence in retrospective studies, necessitating the implementation of prospective studies so that an accurate incidence of such ADRs can be known.

The data presented in this paper demonstrate that the adverse reactions to vancomycin remain a serious public health problem, even after five decades of its inclusion in antimicrobial therapy.

Taking into account the methodological differences and characteristics of each population in previous studies, it can be seen that the ability to control the occurrences of these adverse reactions has not evolved much during the last 20 years.

## Conclusions

Despite the limitations of this study design, small sample size and period of realization (2003-2004), especially regarding the generalization of the results, this study allowed a profile to be drawn showing the reality of the use of vancomycin in a quaternary university hospital with high demand. In assessing the prevalence of adverse reactions to vancomycin it was possible to show that it is still the case that many are not even perceived and even less reported. A larger study, including a more diverse study sample, is necessary in order to replicate the findings and overcome the limitations of the present study. It is also necessary to conduct a multiple variable analysis [31] to disclose the possible interrelations between the different exposure variables pointed by this study, becoming possible to select groups of patients whose vancomycin treatment should be more carefully monitored. Despite the occurrence of ADRs due to vancomycin use, it is an indispensable antibiotic to treat a broad type of severe infections. However the healthcare professionals should be aware of the possibility of vancomycin ADR, emphasizing the importance of pharmacovigilance activities, including an active search for adverse reactions.

## Competing interests

The authors declare that they have no competing interests.

## Authors' contributions

DSM contributed to the data collection process and contributed to the writing of the manuscript. MGRS contributed to the data collection. GH contributed to the data analyses. BLAF contributed to the data collection process. HC and CRR participated in the work coordination and helped to draft the manuscript. VPS and LMC designed the study, supervised data collection, contributed to data interpretation, and critically revised the manuscript for intellectual content. All authors approved the final version of the manuscript.
